# Supplementary figures and images for: Automatic nutrient estimator: distributing nutrient solution in hydroponic plants based on plant growth
Source: PeerJ Comput Sci. 2024 Feb 23;10:e1871. doi: 10.7717/peerj-cs.1871 (PMC10909207; doi:10.7717/peerj-cs.1871)

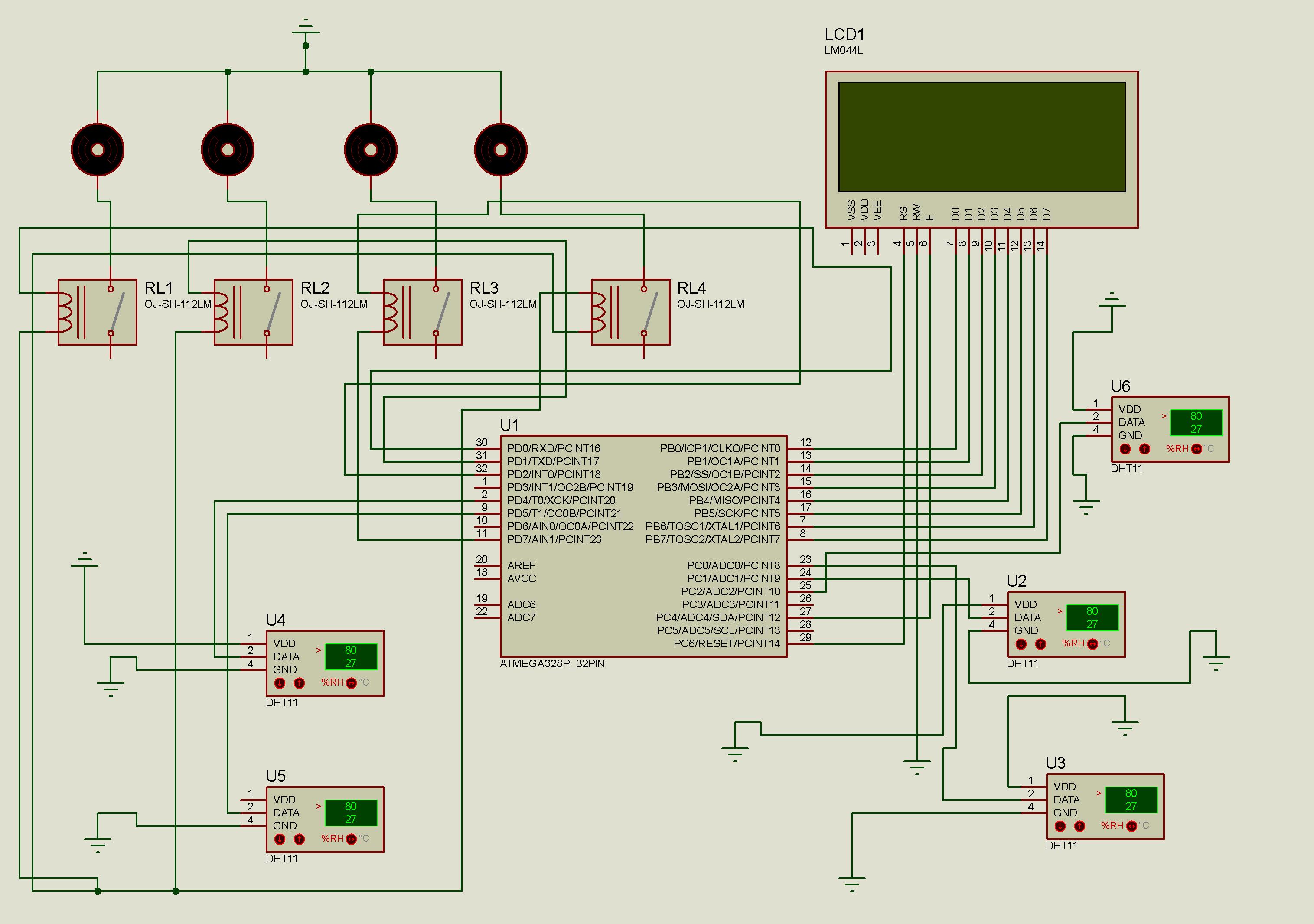

Supplement: Supplemental Information 1 [file peerj-cs-10-1871-s001.jpg]
